# Development and validation of a real-time AI model for differentiating benign and malignant gastric ulcers : a multicenter retrospective study

**DOI:** 10.1186/s12876-026-04848-9

**Published:** 2026-05-04

**Authors:** Yibo Tan, Yongjun Wu, Mei Yang, Yan Li, Xiaofei Bi, Song He, Zhihang Zhou, Junyu Lu

**Affiliations:** 1 Department of Gastroenterology, The Second Affiliated Hospital of Chongqing Medical University, No. 76, Linjiang Road, Yuzhong District, Chongqing, People’s Republic of China; 2 College of traffic and transportation, Chongqing Jiaotong University, Chongqing, People’s Republic of China; 3 Department of Gastroenterology, The Third People’s Hospital of Chengdu, The Affiliated Hospital of Southwest Jiaotong University, Chengdu, People’s Republic of China; 4 Department of Gastroenterology, Chongqing Kaizhou District People’s Hospital, Chongqing, People’s Republic of China; 5 Department of Gastroenterology, Chongqing University Three Gorges Hospital, Chongqing, People’s Republic of China

**Keywords:** Artificial intelligence, Gastric ulcer, Benign and malignant differentiation, Deep learning, Endoscopic diagnosis

## Abstract

**Aim:**

To develop and validate a deep learning-based AI system for the dynamic, real-time differentiation of benign and malignant gastric ulcers during endoscopy, with the goal of enhancing diagnostic precision.

**Methods:**

This was a multicenter, retrospective study collecting endoscopic images and videos from four tertiary hospitals in China. An improved YOLOv8 model, incorporating an illumination attention module, was developed for real-time instance segmentation and classification. The dataset comprised 9,820 benign ulcer images, 1,727 malignant ulcer images, and 15,791 normal mucosa images, split into training, testing, and validation sets at an 8:1:1 ratio. Performance was evaluated based on precision, recall, specificity, and processing latency.

**Results:**

On the validation set, the AI model achieved an overall precision, recall, and specificity of 0.91, 0.91, and 0.95, respectively. For malignant ulcer recognition specifically, the precision, recall, and specificity were 0.90, 0.91, and 0.99. The model demonstrated strong real-time performance with a latency of 8.84 ms per frame and a processing speed of 113 frames per second.

**Conclusion:**

The developed AI model enables accurate, real-time discrimination between benign and malignant gastric ulcers during endoscopy. It holds potential to augment clinical decision-making, standardize diagnostic quality, and optimize biopsy strategies.

**Supplementary Information:**

The online version contains supplementary material available at 10.1186/s12876-026-04848-9.

## Introduction

Gastric ulcer, one of the more common types of peptic ulcers, refers to a condition in which the gastric mucosa is eroded by gastric acid and pepsin, resulting in a breach that extends beyond the muscularis mucosae [1].As a prevalent disease of the digestive system, clinical emphasis is placed on distinguishing between benign and malignant forms of gastric ulcer. Malignant gastric ulcer, also referred to as ulcerative gastric cancer, represents the most frequent pathological manifestation of gastric cancer. Gastric cancer ranks among the top five most common malignant cancers globally and is associated with a high mortality rate. The survival rate for gastric cancer in most regions of the world is approximately 20%, whereas the five-year survival rate for early-stage gastric cancer can reach 90% [2–4].Achieving early and accurate diagnosis of ulcerative gastric cancer poses significant challenges. For instance, there is considerable overlap in symptoms and endoscopic findings between benign ulcers and early gastric cancer. Additionally, following treatment with potent acid-suppressive medications, gastric cancer patients may experience relief from abdominal pain, and the ulcer may shrink or partially heal [5, 6].

Endoscopy with biopsy remains the gold standard for the diagnosis and differential diagnosis of gastric ulcers. However, it has certain limitations: First, the endoscopic assessment of ulcer nature is subjective and heavily reliant on the operator’s experience. Endoscopists primarily diagnose malignant gastric ulcers based on morphological features, such as an irregular, nodular ulcer base, atypical shape, foul and thick exudate, and rough or raised margins. Nevertheless, some benign ulcers with a long history can also exhibit macroscopic features characteristic of malignancy under endoscopy [7],and one study demonstrated that the sensitivity of endoscopy for diagnosing gastric ulcers is 92% [8].Furthermore, a significant issue is the high miss rate associated with endoscopic examination. Several studies have reported that the proportion of undetected gastric cancers reaches 9.8% to 25.8%. A detailed analysis indicated that 73% of these missed cases were attributable to endoscopist error [9, 10].Second, while guidelines recommend obtaining biopsies from lesions with high-risk features identified under white-light imaging, the biopsy rates among endoscopists vary considerably, ranging from 22.4% to 52.9%. Concurrently, as the number of biopsies increases, so does the number of histologically negative biopsies from endoscopically normal-appearing mucosa, imposing an unnecessary burden on both patients and healthcare systems [11].Third, the accuracy of biopsy is dependent on the number and location of the samples obtained. Endoscopic biopsy is an invasive procedure that can cause mucosal damage and bleeding. Repeated biopsies or taking multiple samples over a large area may even lead to submucosal fibrosis [12, 13].

We aim to enhance the real-time endoscopic diagnostic accuracy for ulcer characterization. Emerging technologies offer promising avenues to achieve this goal. Artificial intelligence (AI) has demonstrated considerable potential in auxiliary diagnosis: in recent years, AI models based on deep learning (DL) have made significant strides in medical image analysis. Current studies have indicated the applicability of AI models in automatically assessing the malignant potential of gastric ulcer images. However, most of these studies rely on single-center data and focus on classifying gastric conditions (e.g.: normal mucosa, benign ulcers, malignant ulcers) based on individual static images. This approach diverges from the clinical reality, where endoscopists make diagnoses through continuous observation and holistic assessment of the entire lesion [14–17]. Therefore, we plan to develop an AI system using multi-center data to identify gastric ulcer lesions in real-time during endoscopy. This system will perform benign versus malignant classification and provide pixel-level delineation of the lesions, thereby improving the accuracy of ulcer identification .

## Materials and methods

### Study design

**Fig. 1:**
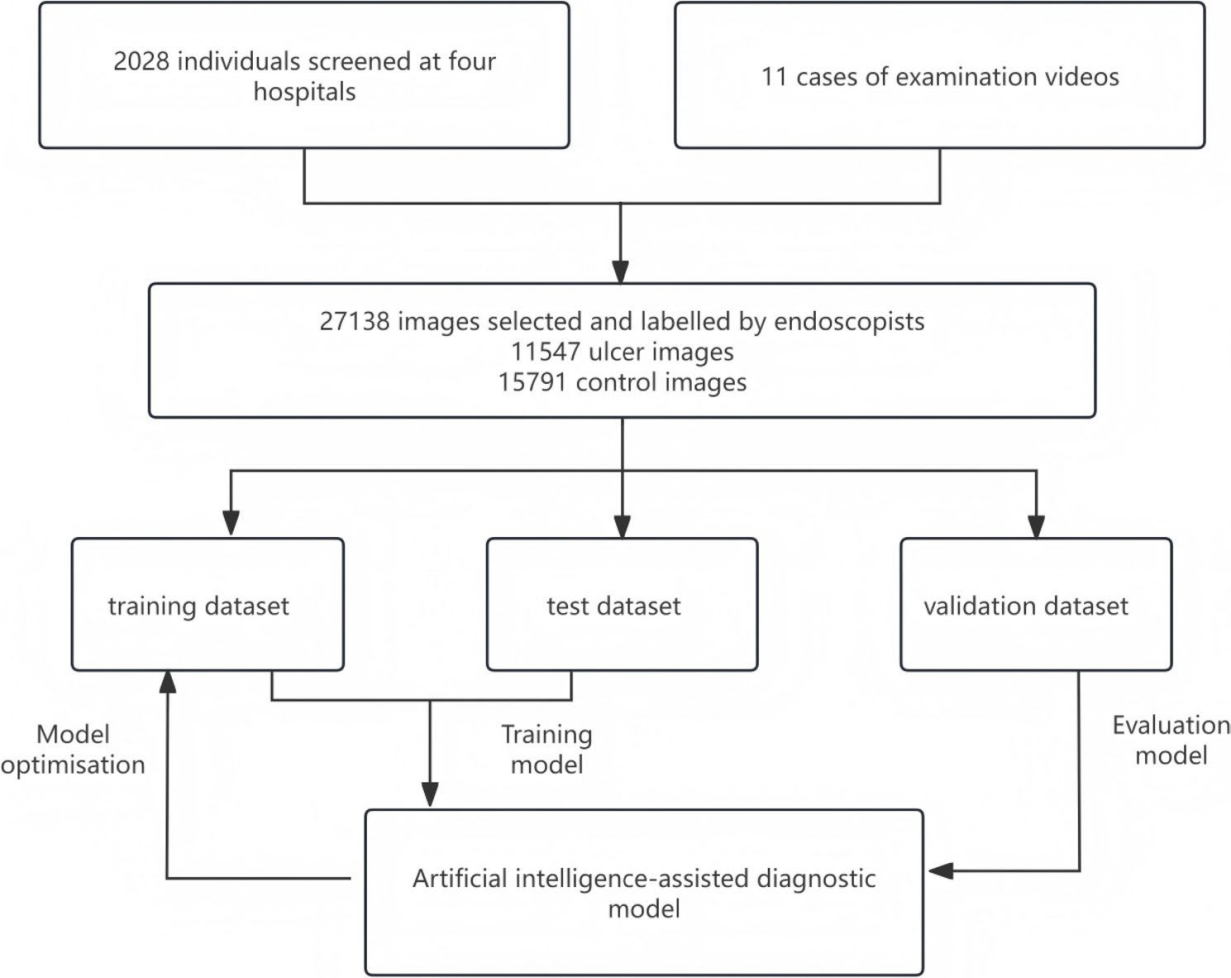
Flowchart of the study design

A brief summary of the study design is shown in Fig. 1. This is a multicenter, retrospective study. All endoscopic images and videos used in this research were retrospectively collected from patients who underwent gastroscopy during routine clinical practice between January 1, 2021, and April 30, 2025, at four tertiary care hospitals in China. The participating hospitals are as follows: The Second Affiliated Hospital of Chongqing Medical University (Chongqing, China), Chongqing University Three Gorges Hospital (Chongqing, China), Chongqing Kaizhou District People’s Hospital (Chongqing, China), and Chengdu Third People’s Hospital (Chengdu, China). All images and videos were captured using standard endoscopes (GIF-Q260, GIF-H260, GIF-H290, Olympus Medical Systems Co. Ltd and HD-500, HD-550, SonoScape Biomedical Technology Co., Ltd). The study was approved by the respective Institutional Review Boards and was conducted in accordance with the Declaration of Helsinki. The requirement for informed consent was waived by the Institutional Review Boards due to the retrospective nature of the study and the use of fully anonymized data.

### Datasets and preprocessing

**Fig. 2:**
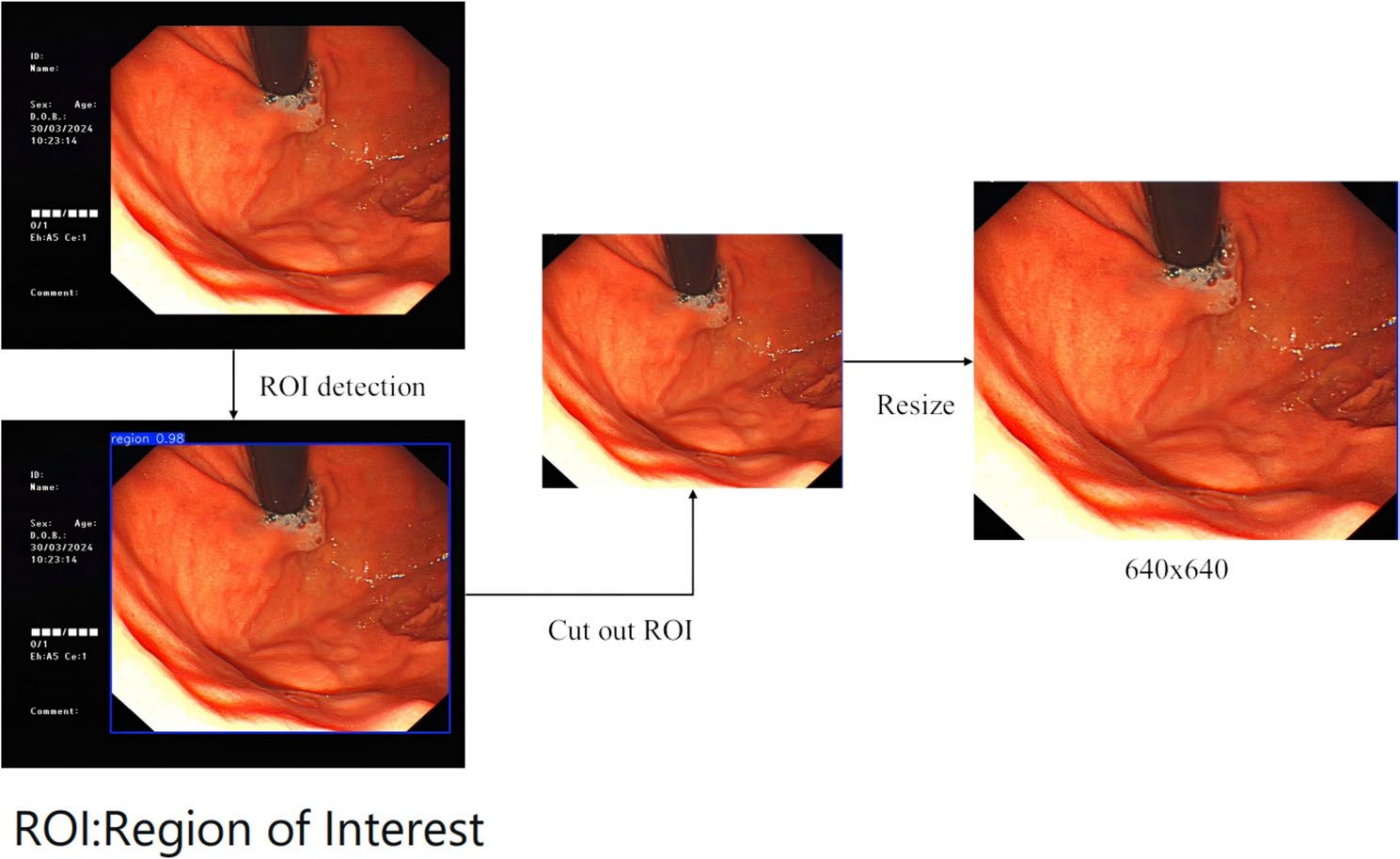
Image preprocessing

The inclusion criteria for images were as follows: (1) Patients aged over 18 who underwent gastroscopy and were histologically diagnosed with benign or malignant ulcerative lesions according to the World Health Organization (WHO) classification; (2) Endoscopic images without ulcerative lesions were included as the “normal gastric mucosa” dataset. The exclusion criteria were: (1) Cases where pathological diagnosis recommended further investigations (such as immunohistochemistry) for definitive characterization, but the patients did not complete these tests; (2) Endoscopic image quality insufficient for reliable endoscopic diagnosis; (3) Inadequate pathological diagnosis due to issues in tissue sampling, sectioning, or staining. Following data curation, the study dataset comprises static images from 2,028 subjects and image frames extracted from 11 additional gastroscopy videos. In total, the dataset contains 9,820 benign ulcer images, 1,727 malignant ulcer images, and 15,791 normal background images, amounting to 27,338 images. A detailed breakdown of the number and distribution of images across categories is provided in Supplementary Tables 1 and 2.All images (including frames extracted from videos) were partitioned at the image level into training, testing, and validation sets in an 8:1:1 ratio. To mitigate the risk of data leakage, we implemented a rigorous data curation process. Specifically, only images exhibiting significant morphological and optical differences were included. Furthermore, experienced endoscopists [18] manually reviewed and removed highly similar or overlapping images of lesions from the same patient. During preprocessing, an object detection model was trained to automatically extract the Region of Interest (ROI) from the output images. This ROI was then used for cropping non-clinical regions, thereby preventing the model from learning patient-specific features unrelated to the lesion itself. The overall procedure is illustrated in Fig. 2. All final images were output in JPG format.

### Building of AI model

This study employed a lightweight YOLOv8 model to construct a real-time gastric ulcer instance segmentation system, capable of simultaneously classifying gastric ulcers and segmenting ulcerous regions. To mitigate interference from varying illumination conditions during endoscopic examinations, an Illumination-Aware Attention module was integrated into the original YOLOv8 architecture. The model was developed using the Python programming language and the PyTorch deep learning framework, with training conducted on three NVIDIA GeForce RTX 3080 Graphics Processing Units (GPUs), each equipped with 24 GB of memory. More detailed methodological information can be found in Supplementary Figs. 1 and 2.

### Model evaluation and statistical methods

#### Classification performance evaluation

Based on the validation set, an N×N matrix (*N* = 3: normal mucosa, benign ulcer, malignant ulcer) was constructed. The columns of the matrix represent the categories marked by the pathological gold standard, and the rows represent the categories predicted by the model. The corresponding precision, specificity, and recall were calculated.

#### Real-time evaluation

Latency per Frame: The time required to process a single image from input to output (ms).

## Results

**Fig. 3:**
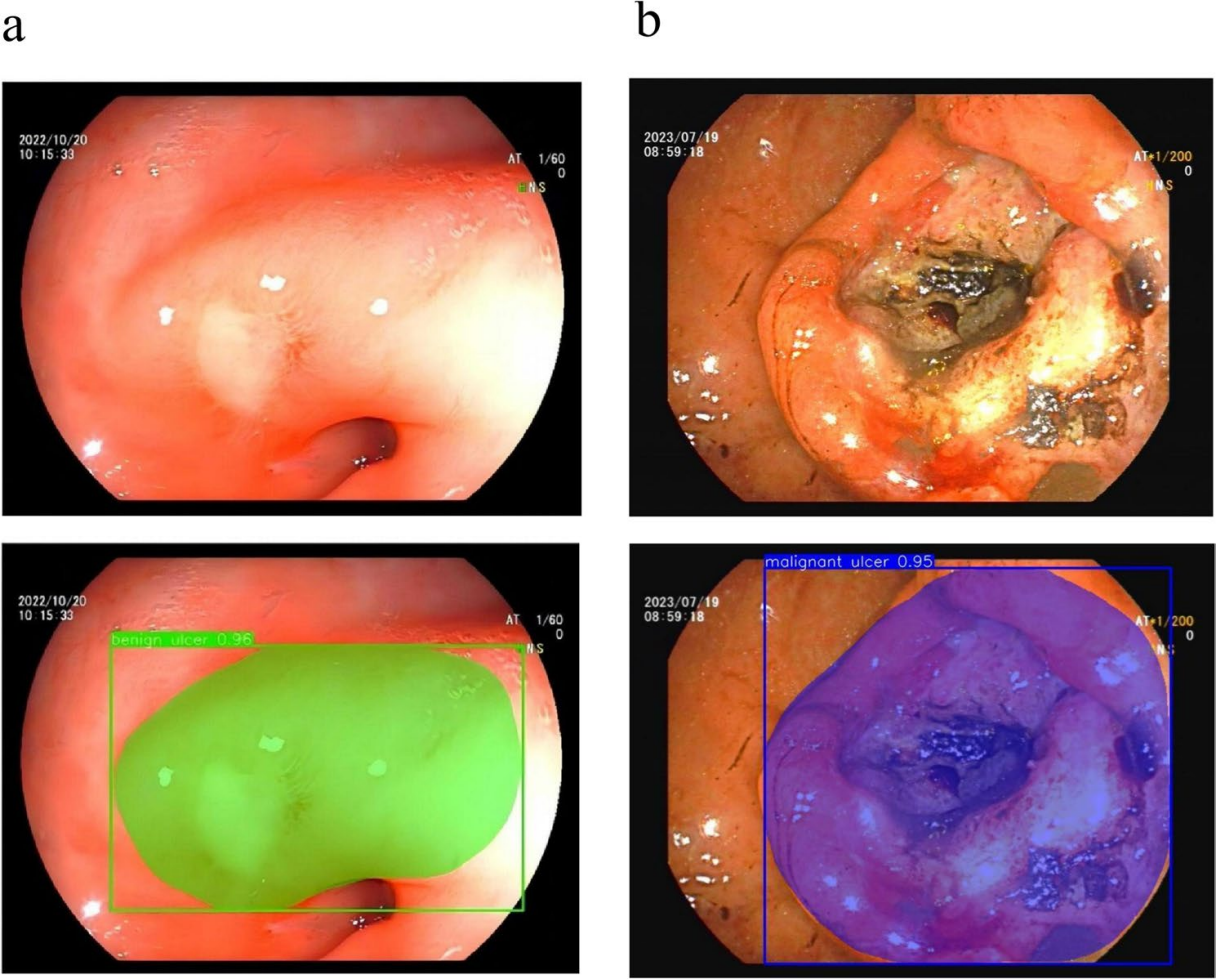
Visualization of the model. **a** an example of a benign ulcer is shown in green and the diagnostic probability outputs is 0.96. **b** an example of malignant ulcer is shown in purple and the diagnostic probability outputs is 0.97

When the AI model detects gastric ulcer lesions from the input data of the validation image, the AI model will output the disease name (benign ulcer or malignant ulcer) and its probability score (range 0–1). The higher the probability score, the higher the confidence in the diagnosis of the model, and the detected lesions will be outlined on the image. Meanwhile, the heat map provided by Gradient-weighted class activation mapping (Grad-CAM) indicates that our model makes reasonable and objective judgments based on specific parts of the image, similar to how endoscopists diagnose lesions. Representative cases are given in Fig. 3 and Figure S3 .

**Fig. 4:**
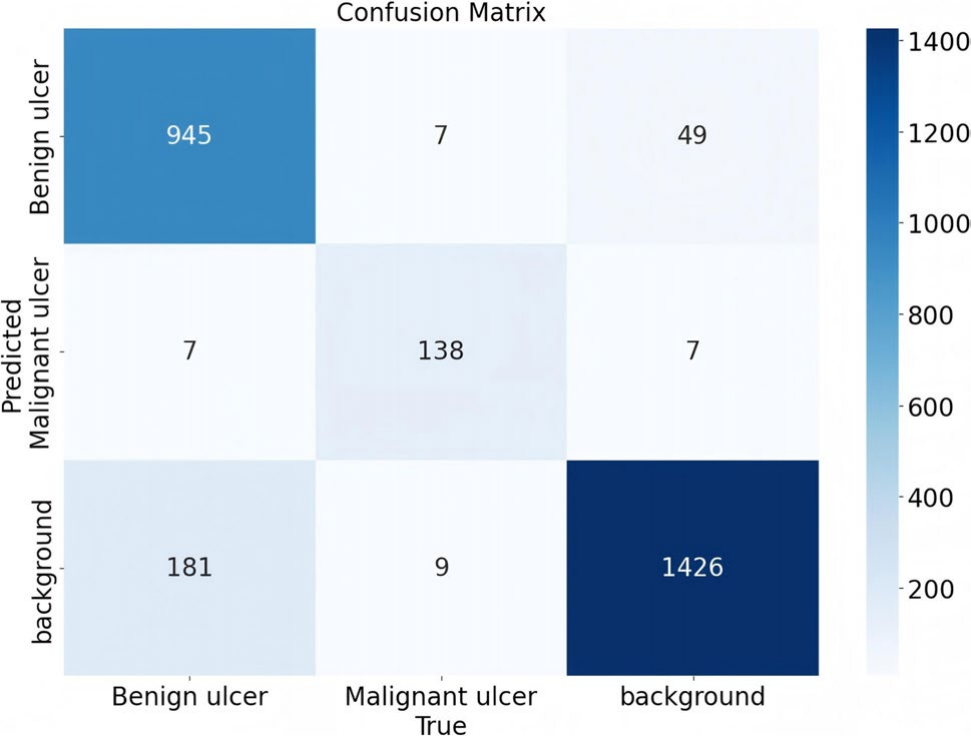
Confusion matrix for the AI model in the validation set

The actual number of pictures in the validation set was 2769, among which the number of lesions of normal gastric ulcer, benign gastric ulcer, and malignant gastric ulcer was 1482, 1133, and 154 respectively. Table 1 shows that the precision rate, recall, and specificity of artificial intelligence in lesion recognition are 0.91, 0.91, and 0.95, among which the precision rate, recall, and specificity of benign lesion recognition are 0.83, 0.94, and 0.89, and the precision rate, recall, and specificity of malignant lesion recognition are all 0.90, 0.91, and 0.99. The more detailed data are presented in Fig. 4.

**Table 1:** A summary of detection performance of the AI model

	Precision	Recall	Specificity
Benign ulcer	0.83	0.94	0.89
Malignant ulcer	0.90	0.91	0.99
Background	0.96	0.88	0.95
All	0.91	0.91	0.95

- 3.3 To evaluate the clinical real-time performance of the model, we recorded the throughput on the validation set. Table 2 shows the speed of artificial intelligence in lesion recognition and processing.

**Table 2:** A summary of real-time performance of the AI model

Number	Time (s)	Speed (fps)	latency (ms)
2713	24	113	8.84

## Discussion

Distinguishing between benign and malignant gastric ulcers is critical for timely and effective clinical intervention. Although endoscopy combined with biopsy remains the gold standard, it is constrained by operator dependency, the potential for missed diagnoses, and the burden on healthcare resources. The advent of AI offers a novel approach to augmenting endoscopic diagnosis by providing more objective and real-time decision-making support. In this multicenter retrospective study, we developed and validated a deep learning-based AI model for the differential diagnosis of benign and malignant gastric ulcers. The proposed model achieved superior performance in the validation set, yielding an overall precision, recall, and specificity of 0.91, 0.91, and 0.95, respectively. Notably, in identifying malignant ulcers, the model demonstrated a precision of 0.90, a recall of 0.91, and a specificity of 0.99. Furthermore, the system exhibited robust real-time processing capabilities, achieving a per-frame processing latency of 8.84 ms and a throughput of 113 fps.

Our study introduces several pivotal technical advancements that distinguish it from prior studies. First, by employing a modified YOLOv8 architecture, our model transcends the simple classification of static images to achieve instance segmentation. This signifies that the AI can not only classify the nature of the lesion but also precisely delineate its boundaries at the pixel level. Second, to enhance the model’s robustness within complex endoscopic environments, we integrated a custom-designed Illumination-Aware Attention Module into the YOLOv8 framework. Through feature channel normalization and a differentiable gating mechanism, this module dynamically fuses the original features with the normalized ones, thereby effectively suppressing common artifacts in endoscopic imaging, such as uneven illumination and specular reflections [19, 20], thereby enhancing the model’s reliability across diverse clinical settings. Third, given that the heterogeneity and diversity across datasets can significantly impact AI performance, datasets that adequately capture real-world variability are of paramount importance [21, 22].The datasets were curated from four tertiary healthcare institutions, augmented by extracting frames from authentic endoscopic video recordings. This frame-extraction methodology empowers the model to discern subtle pathomorphological characteristics frequently underappreciated in static imaging. For instance, exudates on benign ulcers may partially detach following air insufflation, whereas those in malignant ulcers typically exhibit more tenacious adherence due to infiltrative growth patterns. These observed morphological and adherence characteristics emulate aspects of the clinical reasoning process employed by endoscopists during examination, thereby enhancing the ecological validity of our dataset in representing real-world clinical scenarios. Furthermore, by training on diverse static images extracted from video sequences, the model has acquired processing and recognition capabilities during dynamic endoscopic observation (a specific example can be found in Video 1), thereby enhancing its adaptability in real-world clinical scenarios.

The diagnostic accuracy and real-time performance demonstrated by our AI model hold significant potential for clinical application. First, based on the classification performance metrics derived from the validation set, these indicators are comparable to the performance reported for expert endoscopists in similar studies, and significantly outperform those of junior endoscopists [15, 23, 24]. We hypothesize that our AI model may possess expert-level diagnostic potential. Direct, head-to-head comparative studies are necessary to validate this hypothesis. Second, the model’s proficiency in accurately identifying and segmenting malignant ulcers holds the potential to assist clinicians in guiding biopsy strategies, by directing them to prioritize sampling from the most suspicious regions and potentially reducing unnecessary biopsies of benign lesions or normal mucosa. Furthermore, in the context of real-time diagnosis and treatment within gastrointestinal endoscopy, clinical decisions are made within stringent time windows due to the cumulative anesthetic risks and spatial constraints of endoscopic manipulation [25, 26]. Specifically, when an endoscopist identifies a suspicious ulcerative lesion, they must make a series of critical decisions in a very short period. Consequently, the system must possess efficient image processing capabilities. We recorded the processing time for each frame within the data stream, and our results demonstrate that our model exhibits excellent real-time performance, fulfilling the requirements for future dynamic identification [27].

Although deep learning architectures like YOLOv8 offer excellent real-time performance, which is crucial for endoscopic applications, they inherently present a “black-box” challenge. To address this, we implemented a multi-faceted approach. Firstly, we introduced Gradient-weighted Class Activation Mapping (Grad-CAM) techniques as illustrated in Supplementary Figure S3 to provide interpretability by highlighting the specific image regions the AI prediction focuses on. These visualization results confirm that the model attends to clinically relevant features, such as irregular ulcer borders and basal characteristics, rather than random background noise. Secondly, the incorporation of an illumination-aware attention module and training on dynamic video frames bolsters the model’s robustness and reduces its reliance on spurious correlations, further mitigating concerns about “random effects.” By dissecting the AI model’s diagnostic logic through a combination of post-processing and pre-processing methods, we have concretized abstract theories, enhanced the interpretability of AI diagnoses, and consequently improved diagnostic accuracy and consistency [28].

Despite achieving initially encouraging results, several limitations warrant acknowledgment. Firstly, the retrospective design of the study is inherently susceptible to selection bias. Secondly, although the multi-center data source enriched sample diversity, prior research indicates that multi-center data may lead to greater heterogeneity in negative cases and less heterogeneity in positive cases, potentially attenuating the ability to identify positive lesions—a trend consistent with our findings [29]. Thirdly, despite implementing rigorous manual deduplication and multimodal differentiation, partitioning the dataset at the image level to simulate real-time video stream input introduces a potential risk of data leakage. Future validation using independent, prospective, patient-level cohorts is necessary to definitively ascertain the model’s generalizability. Fourth, misclassification of malignant ulcers is a key issue. Sixteen cases of malignant ulcers were misclassified. This false negative represents a more serious clinical risk and may lead to delay in diagnosis and treatment. We revisited our validation set and found that atypical ulcer morphology and additional interference in endoscopic images may be the cause of misdiagnosis, as detailed in the Supplementary figure S4. Fifth, the potential for the AI system to optimize biopsy strategies remains hypothetical. Direct clinical trials are needed to validate its impact on actual biopsy decisions and patient outcomes.

In conclusion, this study successfully developed an artificial intelligence model capable of dynamically distinguishing between benign and malignant gastric ulcers in real-time, leveraging multi-center data and an enhanced YOLOv8 architecture. The model demonstrated excellent performance in retrospective image and video detection. Despite inherent data and technical limitations, it shows promising potential to transform traditional endoscopic diagnostic workflows. Future research should focus on multimodality (such as incorporating molecular pathology and language text information [30, 31], prospective validation, and the establishment of an ethical framework, in order to facilitate its comprehensive transition from technological exploration to routine clinical application.

## Supplementary information

Supplementary Material 1.

Supplementary Material 2.

## Data Availability

The datasets used during the current study are available from the corresponding author on reasonable request.

## Abbreviations

AI: Artificial intelligence
DL: Deep learning
WHO: World Health Organization
ROI: Region of Interest
YOLOv8: You Only Look Once Version 8
Grad-CAM: Gradient-weighted class activation mapping
FPS: Frames per second
