# Supplementary material for: Development and validation of a real-time AI model for differentiating benign and malignant gastric ulcers : a multicenter retrospective study
Source: BMC Gastroenterol. 2026 May 4;26:378. doi: 10.1186/s12876-026-04848-9 (PMC13285405; doi:10.1186/s12876-026-04848-9)
Supplement: Supplementary file 1 — Supplementary Material 1. [file 12876_2026_4848_MOESM1_ESM.docx]

**Appendix**

Table S1 Image data distribution from each participating hospital

|  | Center1 | Center2 | Center3 | Center4 | Whole datasets |
| --- | --- | --- | --- | --- | --- |
| Benign ulcer | 1456（495）* | 1484（272） | 4806（668） | 517（143） | 8263（1578） |
| Malignant ulcer | 480（140） | 239（25） | 724（96） | 8（2） | 1451（263） |
| Background | 10353（187） | / | / | / | 10353（187） |
| Overall | 12289（822） | 1723（297） | 5530（764） | 525（145） | 20067（2028） |

Center 1: The Second Affiliated Hospital of Chongqing Medical University; Center 2: The Chongqing University Three Gorges Hospital; Center 3: The People's Hospital of Kaizhou District;Center 4: The Third People's Hospital of Chengdu;

*The numbers represent the number of images, and the content in parentheses represents the corresponding number of patients.

The pathological types of malignant tumors in the dataset include gastric cancer (including gastric adenocarcinoma and signet ring cell carcinoma): 260 cases. Malignant lymphoma: 2 cases. Other malignant tumors: 1 case (mixed adenoneuroendocrine tumor).

Table S2 Video data distribution

|  | Benign ulcer | Malignant ulcer | Background | Whole dataset |
| --- | --- | --- | --- | --- |
| the number of images | 1557 | 276 | 5438* | 7271 |

|  | Benign ulcer | Malignant ulcer | Background | Whole dataset |
| --- | --- | --- | --- | --- |
| number of patients | 7 | 3 | 1 | 11 |

* It should be noted that in the process of extracting frames from the real video, for the images generated in non-disease area, we classified them into the background dataset; therefore, the 5,438 images in the background dataset were actually obtained from 11 videos.

The pathological types of the three cases of malignant ulcers were all gastric adenocarcinoma.

Figure S1 Overview of the AI model conceptual framework


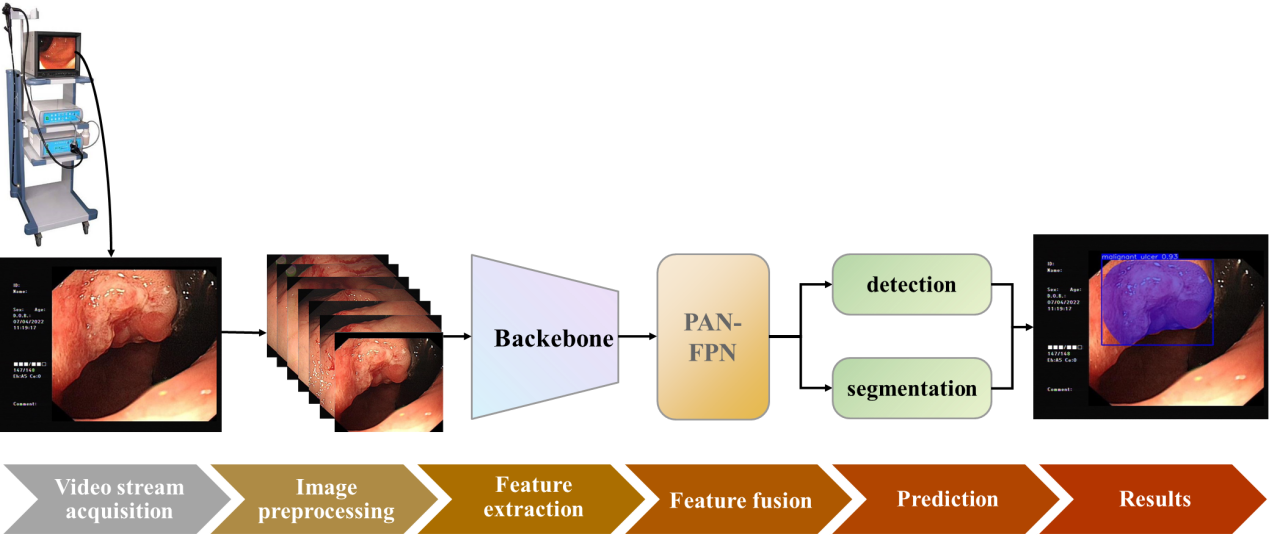


PAN-FPN: Path Aggregation Network and Feature Pyramid Networks

a. The model construction primarily involves the following steps: video and image acquisition, image preprocessing, feature extraction, multi-scale feature fusion, model inference and result prediction, and result output.

b. To mitigate the impact of illumination variations, a designed illumination attention module is incorporated into the original YOLOv8 architecture. The overall model structure is illustrated in Figure b.


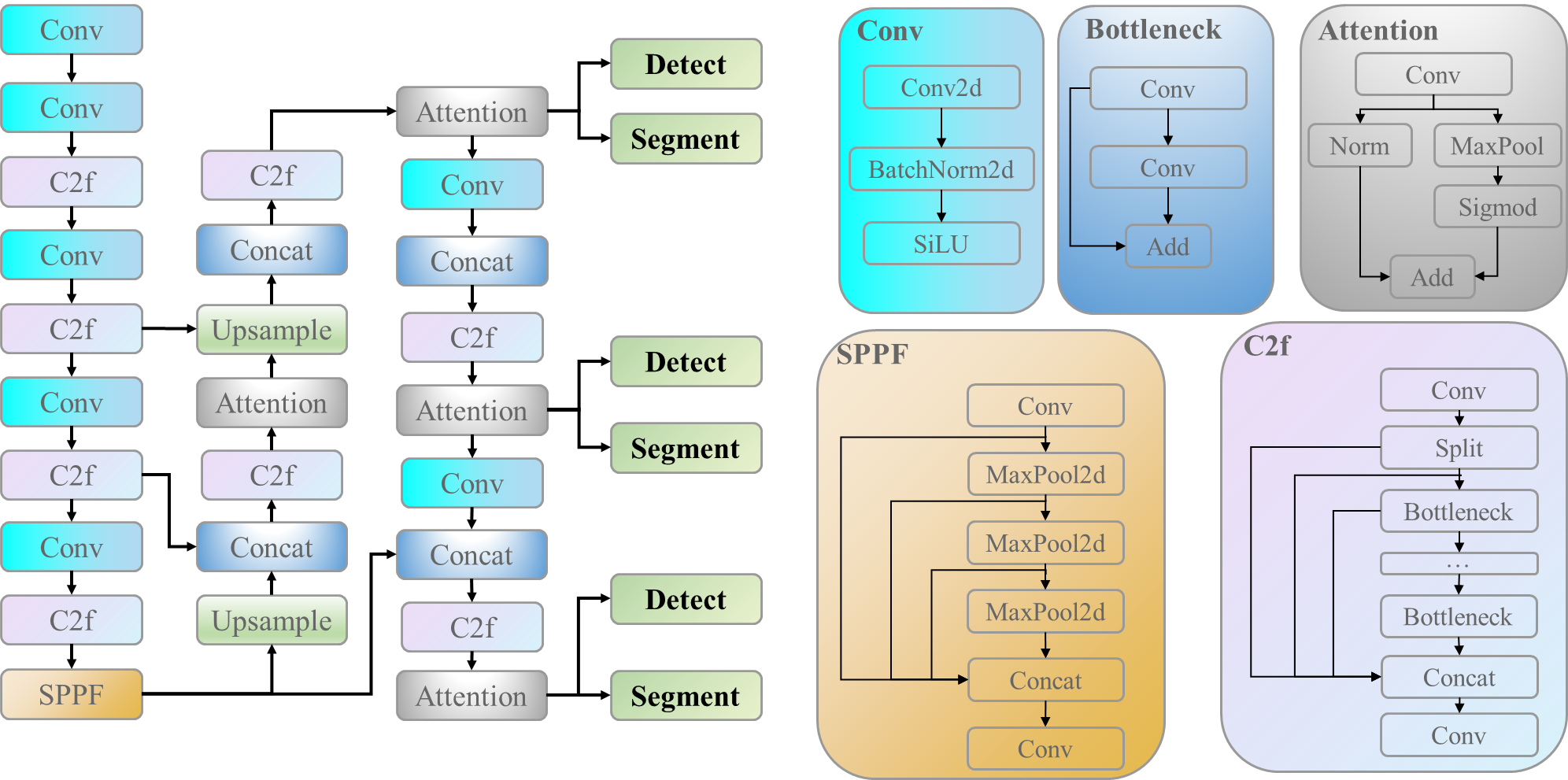


Conv, convolutional layer. C2f, CSP bottleneck with 2 convolutions. SPPF, Spatial Pyramid Pooling Fast. Conv2d, two-dimensional convolutional layer. BatchNorm2d, two-dimensional batch normalization layer. SiLU, Sigmoid-Weighted Linear Unit. MaxPool2d, max pooling layer. Sigmod, Sigmoid function. Add, elementwise addition layer. Upsample, upsample layer. Concat, concatenate layer. Split, dimensional split layer. Detect, detection head. Segment, segmentation head. Attention, attention layer.

Specifically, the illumination attention module normalizes the features extracted by the network to reduce the influence of illumination.

Within illumination attention module, channel normalization is first applied to the extracted features to obtain refined representations. The normalization is computed as follows:

$$x'=\frac{x-\mu\left(x\right)}{\sigma\left(x\right)}\gamma+\beta$$

where $x'$ denotes the normalized feature, $\mu\left(x\right)$ and $\sigma\left(x\right)$ represent the mean and variance of the original feature, respectively, and $\gamma$ and $\beta$ correspond to the transformed mean and variance, which are adjusted during the model training process.

Subsequently, a differentiable gating module is employed to efficiently select between the original features and the normalized features along the channel dimension. A series of binary indicators are obtained through pooling and normalization functions. The specific computation is formulated as follows:

$$p_{x}=pooling\left(x\right)$$

$$g_{x}=\frac{p_{x}^{2}}{p_{x}^{2}+\varepsilon}$$

where $p_{x}$ denotes the pooled feature; $g_{x}$ represents the gating channel selection vector; $\varepsilon$ is a small positive constant introduced to prevent division by zero; and refers to the pooling function, for which the max-pooling operation is adopted.

Finally, channel selection is utilized to fuse the original backbone features with the normalized features. The fusion rule is formulated as follows:

$$x_{n+1}=\left(1-g\right)\odot x+g\odot x'$$

where $x_{n+1}$ denotes the fused feature, and indicates channel-wise multiplication.

Figure S2 Training results of the improved the model.


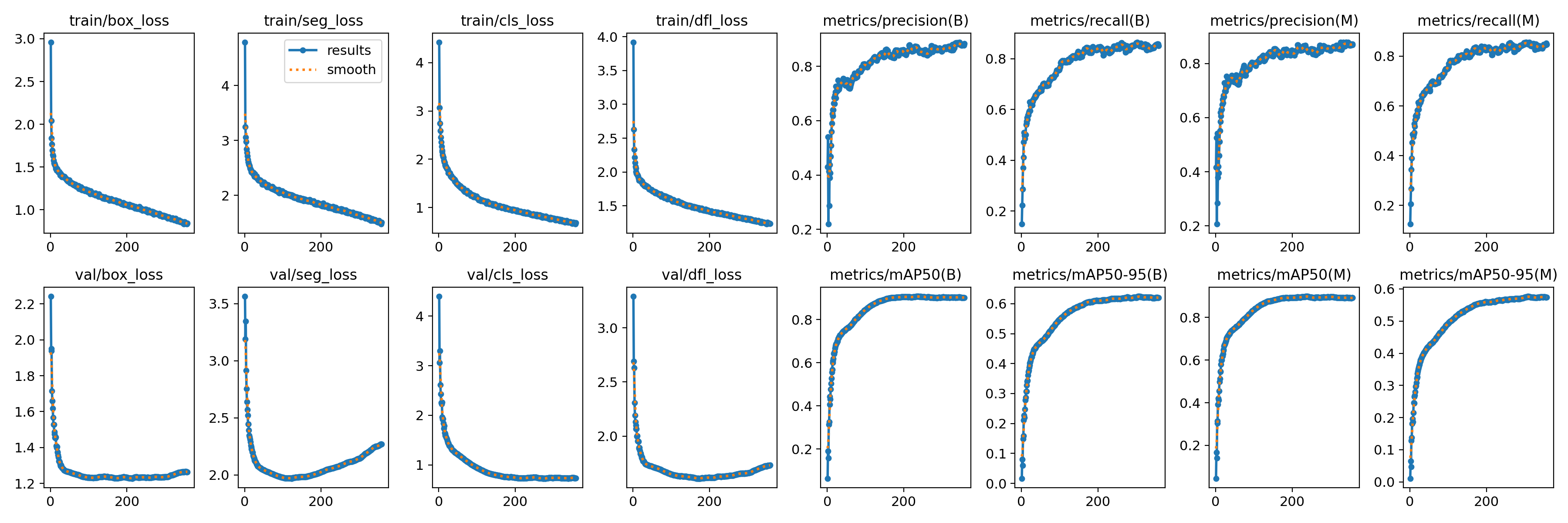


The bounding box localization loss, category classification loss, segmentation loss, and distribution focal loss gradually decrease with increasing iterations on the training set, while on the validation set, each type of loss tends to stabilize after about 300 iterations.

Figure S3 Interpretability of the model

Using heat maps to provide interpretability of artificial intelligence. The highlighted areas in the image indicate the regions that the artificial intelligence pays more attention and the image above is the original endoscopic image, and the corresponding image below is a heat map. We each listed three examples of benign and malignant ulcers.

| benign ulcers |  |  |
| --- | --- | --- |
| 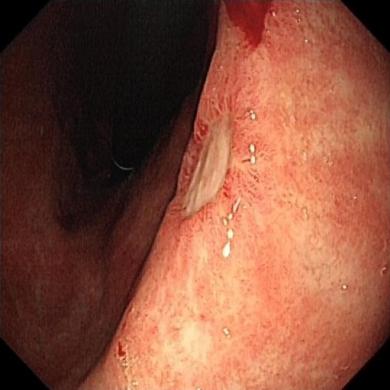 | 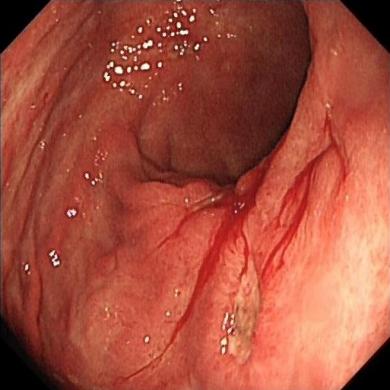 | 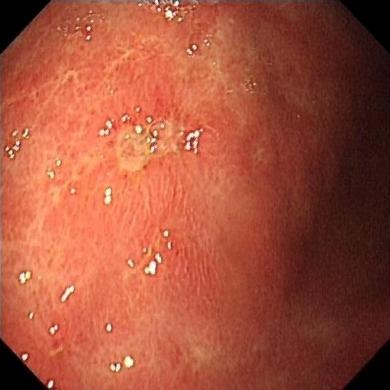 |
| 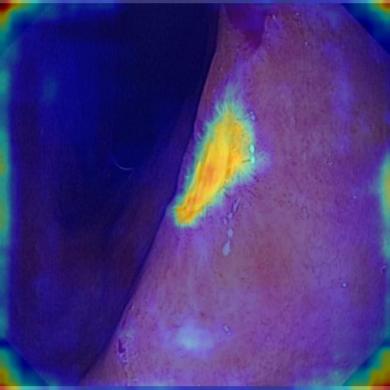 | 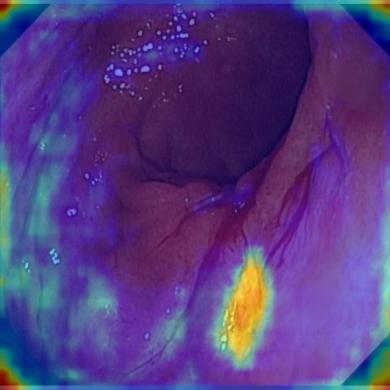 | 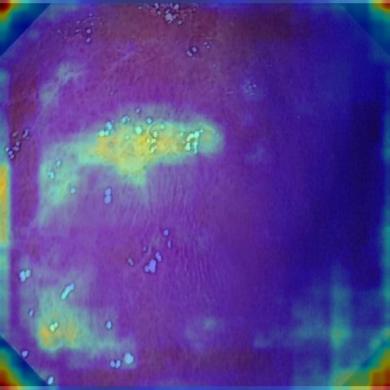 |
| Malignant ulcers |  |  |
| 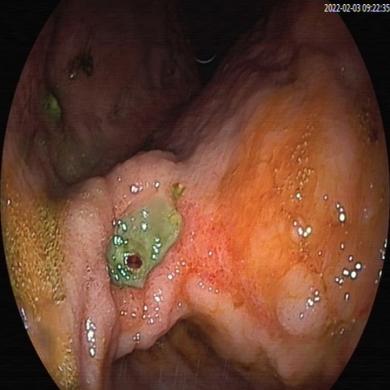 | 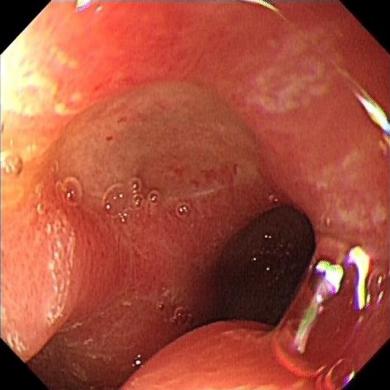 | 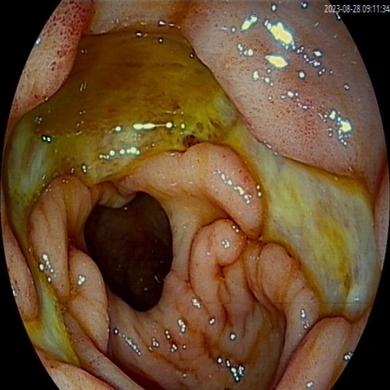 |
| 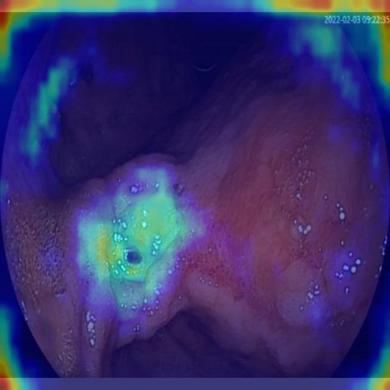 | 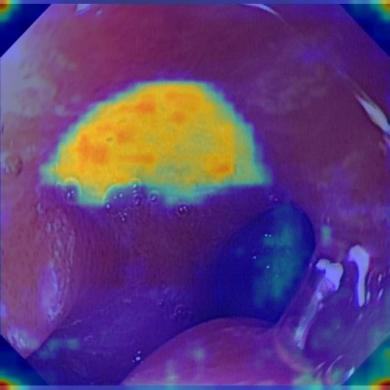 | 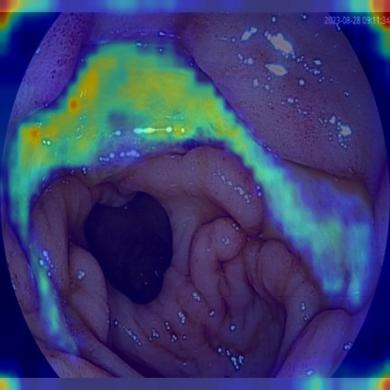 |

Figure S4 Typical misclassification

A.Atypical morphology


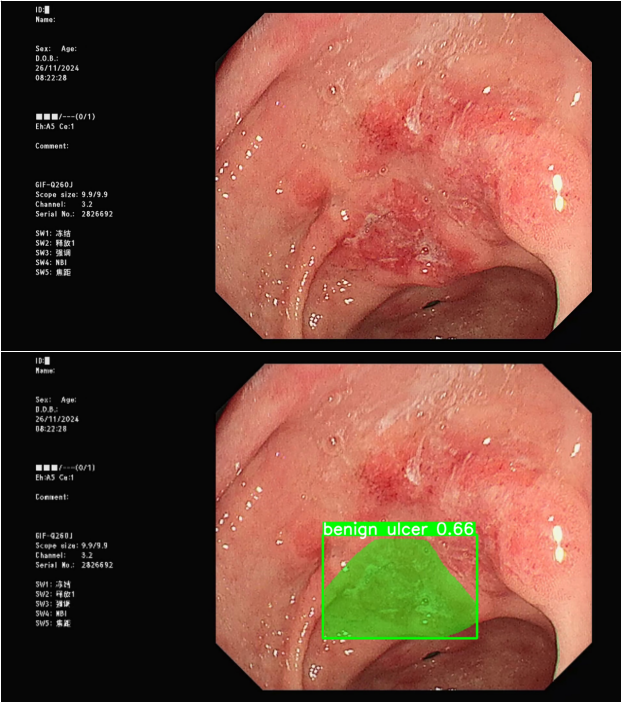


The lesion was selected from a malignant gastric antrum ulcer, which was misdiagnosed as a benign ulcer by the artificial intelligence model. We considered that the lesion in this case was atypical ulcer morphology, and the lesion included both ulcer and healing mucosa, which was not a typical simple ulcer. Therefore, the classification was wrong, but the confidence of artificial intelligence in its classification was also very low, only 0.66.

1. Additional distractions
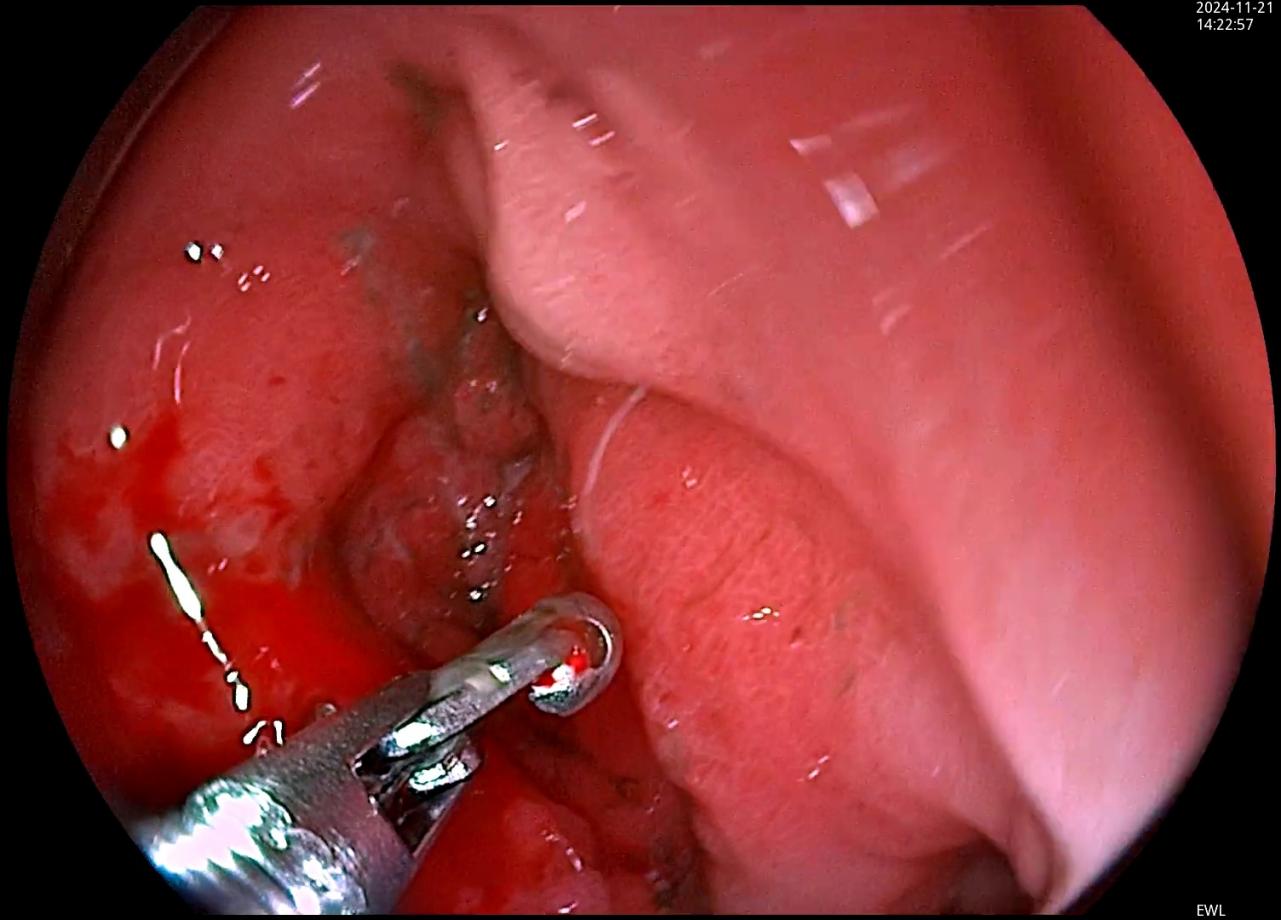


In the following case of a missed malignant gastric antrum ulcer, the image was obtained from a real endoscopy video, and the missed diagnosis was considered to be due to the presence of the biopsy forceps affecting the judgment of the model.
